# Molybdenum Disulfide/Nickel-Metal Organic Framework Hybrid Nanosheets Based Disposable Electrochemical Sensor for Determination of 4-Aminophenol in Presence of Acetaminophen

**DOI:** 10.3390/bios13050524

**Published:** 2023-05-07

**Authors:** Zahra Dourandish, Iran Sheikhshoaie, Shahab Maghsoudi

**Affiliations:** Department of Chemistry, Faculty of Science, Shahid Bahonar University of Kerman, Kerman 76175-133, Iran

**Keywords:** 4-aminophenol, acetaminophen, disposable electrochemical sensor, MoS_2_/Ni-MOF hybrid nanosheets

## Abstract

The toxicity of commonly used drugs, such as acetaminophen (ACAP) and its degradation-derived metabolite of 4-aminophenol (4-AP), underscores the need to achieve an effective approach in their simultaneous electrochemical determination. Hence, the present study attempts to introduce an ultra-sensitive disposable electrochemical 4-AP and ACAP sensor based on surface modification of a screen-printed graphite electrode (SPGE) with a combination of MoS_2_ nanosheets and a nickel-based metal organic framework (MoS_2_/Ni-MOF/SPGE sensor). A simple hydrothermal protocol was implemented to fabricate MoS_2_/Ni-MOF hybrid nanosheets, which was subsequently tested for properties using valid techniques including X-ray diffraction (XRD), field emission-scanning electron microscopy (FE-SEM), energy dispersive X-ray spectroscopy (EDX), Fourier transformed infrared spectroscopy (FTIR), and N_2_ adsorption-desorption isotherm. The 4-AP detection behavior on MoS_2_/Ni-MOF/SPGE sensor was followed by cyclic voltammetry (CV), chronoamperometry and differential pulse voltammetry (DPV). Our experimental findings on the generated sensor confirmed a broad linear dynamic range (LDR) for 4-AP from 0.1 to 600 μM with a high sensitivity of 0.0666 μA/μM and a low limit of detection (LOD) of 0.04 μM. In addition, an analysis of real specimens such as tap water sample as well as a commercial sample (acetaminophen tablets) illuminated the successful applicability of as-developed sensor in determining ACAP and 4-AP, with an impressive recovery rate.

## 1. Introduction

HOC_6_H_4_NH_2_, p-Aminophenol or 4-aminophenol is known as a raw material with wide and varied applications in the manufacture of pharmaceutical products, thermal dyes, black and white photographic developer, antioxidants, polymer stabilizers, petroleum additives, fungicides, herbicides, and insecticides [1,2]. Such extensive applications have inevitably led to large concentrations of 4-AP being introduced into the environment and in particular water sources, which can leave toxic effects due to the presence of structural phenol and aniline. The penetration of 4-AP into the human body can be associated with serious health consequences such as dermatitis, eczema, nephrotoxicity, and teratogenic complications [3,4]. Therefore, some European countries and the United States have recommended that the maximum allowable dose of 4-AP in pharmacy should be up to 50 ppm [5]. Thus, both biochemically and environmentally hazardous 4-AP can contaminate and threaten both environmental resources and the health of life by easily penetrating the skin and membranes of plants.

N-acetyl-p-aminophenol, Paracetamol, Acetaminophen or ACAP, is known as one of the most common antipyretic and analgesic drugs that impact mainly the management of migraine pain, headache, arthritis, cancer pain, back pain, and postoperative pain [6,7,8]. ACAP can also be prescribed to aspirin-sensitive patients, such as those with hemophilia, varicella, and other bleeding conditions [9]. Despite these advantages, ACAP can be partially degraded to 4-AP in pharmaceutical formulations during synthesis or storage. In fact, toxic 4-AP can accumulate in the human body following over-administration of ACAP, leading to some severe diseases such as deformity, renal toxicity, liver toxicity, pancreatitis and skin rash [10,11]. In addition, the environmental accumulation of ACAP and 4-AP excreted is increasingly observed in a variety of water sources. The toxic nature of ACAP and 4-AP underscores the need to achieve an effective approach in their simultaneous determination.

Some of the techniques available in this field are spectrophotometry [12], chemiluminescence [13], high performance liquid chromatography [14], and capillary electrophoresis [15]. Despite the unique advantages of all these techniques, they suffer from some disadvantages such as being time-consuming, having high cost, and the need for precision tools, tedious operations, and boring sample preparation [16]. In the meantime, special attention has been paid to electrochemical measurement approaches due to advantages such as simplicity, simple preparation of specimens and sensors, high accuracy, and fast response [17,18,19,20,21,22,23,24]. Nevertheless, bare electrodes in electrochemical determinations have shown disadvantages such as low sensitivity, poor reproducibility and low electrochemical response [25,26,27,28,29,30,31]. In this context, the potentials of redox peak can be obscured by cross-interference when applying a bare screen printed graphite electrode for sensing ACAP and 4-AP [32]. Therefore, there is a need for a modifier for surface modification of the electrodes to achieve some advantages such as low LOD, broad working range, and high accuracy during analytical applications [33,34,35,36,37,38,39,40,41].

Ultrathin two-dimension (2D) nanosheets (NSs) have been introduced as promising electrode materials because of admirable attributes like huge specific surface area, impressive electronic behaviors, and high mechanical strength [42,43], particularly in the field of nanotechnology [44].

Molybdenum disulfide (MoS_2_) is a layered quasi-2D chalcogenide material, similar to graphite structure. Three atomic layers are found in MoS_2_, including a layer of Mo positioned between two layers of S with the aid of weak van der Waals forces [45,46]. It has a variety of applications in catalysts, lithium batteries, sensors and supercapacitors because of commendable catalytic ability, impressive electron mobility, huge surface area and high electronic density [47,48].

Metal-organic frameworks (MOFs) such as promising crystalline porous materials are formed by binding metal ions or clusters using organic ligands based on coordination chemistry [49,50,51]. Some of the fields of application of MOFs are drug, catalysis, gas separation, magnetism and fluorescence owing to their huge specific surface area, pore structure, functional nature, drug delivery and adjustable architecture [52]. Among these, 2D MOFs have shown special attributes when compared with bulk MOF materials, such as high porosity and nano-sized thickness that predispose excellent electron transfer and rapid mass transport. Unlike the bulk MOFs’ active sites, 2D MOFs show many available active sites on the layers’ surface, thereby facilitating the contact of active sites with substrates and consequently improving the catalytic behavior of MOFs [53,54,55,56]. It should be noted that many nano-sized metal sulfides are adsorbed into MOF NSs as in-situ to generate nano-hybrids. The activity can be improved due to the synergism of all components [57]. We used the synergistic properties of MoS_2_ and Ni-MOF NSs to achieve a facile protocol to fabricating the MoS_2_/Ni-MOF nanohybrid sheets, which was applied to modify the SPGE surface for 4-AP electrooxidation. The findings showed commendable electrocatalytic performance of MoS_2_/Ni-MOF/SPGE for the oxidation/reduction process of 4-AP. In addition, the proposed sensor was employed to voltammetrically detect 4-AP and ACAP in real specimens.

## 2. Experimental Section

### 2.1. Apparatus and Materials

(NH_4_)_6_Mo_7_O_24_•4H_2_O, thiourea, nickel (II) nitrate hexahydrate, terephthalic acid, KOH, N,N-Dimethylformamide (DMF), 4-aminophenol and acetaminophen were purchased from Sigma-Aldrich. All other reagents had analytical grade. Orthophosphoric acid was used to prepare phosphate buffer solution (0.1 M, PBS) as a supporting electrolyte.

An Autolab potentiostat/galvanostat type PGSTAT302N made by Eco Chemie in Netherlands, which has been also provided with the General-Purpose Electrochemical System (GPES 4.9) was applied in each of the electrochemical experiments. The screen-printed graphite electrode (SPGE) was made by Dropsens (DRP-110) in Asturias, Spain. This electrode consists of a graphite working electrode, a silver pseudo reference electrode; and a graphite counter electrode. The solutions pH was adjusted by a pH-meter (Metrohm 710).

FE-SEM images were obtained on a MIRA3TESCAN microscope alongside energy-dispersive X–ray analysis. MoS_2_/Ni-MOF nanosheets structure was characterized using X-ray diffraction patterns (X’Pert Pro X-ray diffractometer, Panalytical, Netherlands) applying Cu/Kα radiation (λ = 1.5418 nm). Then, the Bruker spectrometer (KBr pellets, Tensor-27) made by Germany was utilized to record a FTIR spectrum on the wavelengths 4000 cm^−1^ to 400 cm^−1^. The MoS_2_/Ni-MOF hybrid nanosheets were also examined for their surface area by a BELSORP MINI II Brunauer–Emmett–Teller device (BET) with the Barrett–Joyner–Halenda (BJH) analysis for pore size determination.

### 2.2. Fabrication of MoS_2_

A typical protocol was followed to prepare MoS_2_ NSs [58,59]. Thus, (NH_4_)_6_Mo_7_O_24_·4H_2_O (3 mmol) and thiourea (2.3 g) were poured in deionized water (30 mL) and then heated for 24 h at 200 °C inside a 50-mL Teflon autoclave. The product was thoroughly rinsed with deionized water/ethanol (with a volume ratio of 1:1), and finally dried at 50 °C for six hours under vacuum condition.

### 2.3. Fabrication of MoS_2_/Ni-MOF Hybrid Nanosheets

In situ growth of Ni-MOF on MoS_2_ surface led to the formation of the hybrid NSs of MoS_2_/Ni-MOF. Thus, MoS_2_ (0.32 g) was poured in DMF (15 mL) under 30-min ultra-sonication, followed by adding nickel (II) nitrate hexahydrate (0.87 g, 3 mmol) dispersed in deionized water (10 mL) under another 30-min ultra-sonication. The obtained solution was then slowly appended with DMF solution (10 mL) including terephthalic acid (0.17 g, 1 mmol) and 0.4 M KOH (5 mL). Following another sonication for two hours, the obtained solution was subjected to solvothermal reaction inside the 50-mL Teflon stainless steel autoclave at 120 °C for 24 h. Next, the product was cooled down to room temperature, followed by centrifugation to obtain the precipitate that was subsequently rinsed with deionized water/DMF (with a volume ratio of 1:1) thoroughly, and finally dried at 65 °C for 24 h under vacuum condition.

Moreover, Ni MOF NSs were prepared as control, similar to the method used to produce MoS_2_/Ni MOF hybrid NSs, except for the addition of MoS_2_ prior to solvothermal treatment.

### 2.4. SPGE Modification with MoS_2_/Ni MOF Hybrid Nanosheets

In this step, SPGE was coated by the MoS_2_/Ni MOF hybrid NSs. Thus, 1 mg of MoS_2_/Ni MOF hybrid NSs was dispersed in 1 mL of aqueous solution through a 45-min ultrasonication to prepare a stock solution of the hybrid nanosheets of MoS_2_/Ni MOF. Then, the graphite working electrodes were used to cast 5 µL of the suspension solution of the MoS_2_/Ni MOF nanohybrid. Finally, we put the solvent at room temperature to evaporate to achieve the MoS_2_/Ni-MOF/SPGE.

### 2.5. Preparation of Real Specimens

In order to prepare the real sample of an ACAP tablet (labeled 325 mg), the first five tablets of the ACAP were powdered with a mortar and pestle. Then, 325 mg of this powder was dissolved in 25 mL deionized water under ultra-sonication. Then, in order to remove impurities and fillers in the tablet, the resulting sample was filtered by using filter paper. Then, a specific volume of this solution was transferred to a volumetric flask (25 mL) and diluted with 0.1 M PBS (pH = 7.0). Finally, the standard addition method was used to determine the ACAP and 4-AP content in tablet samples.

Tap water specimens filtrated with a membrane filter and added into 0.1 M PBS (pH = 7). At last, the ACAP and 4-AP contents were measured in the tap water samples using the developed protocol according to standard addition method.

## 3. Results and Discussion

### 3.1. Characterizations

**Figure 1:**
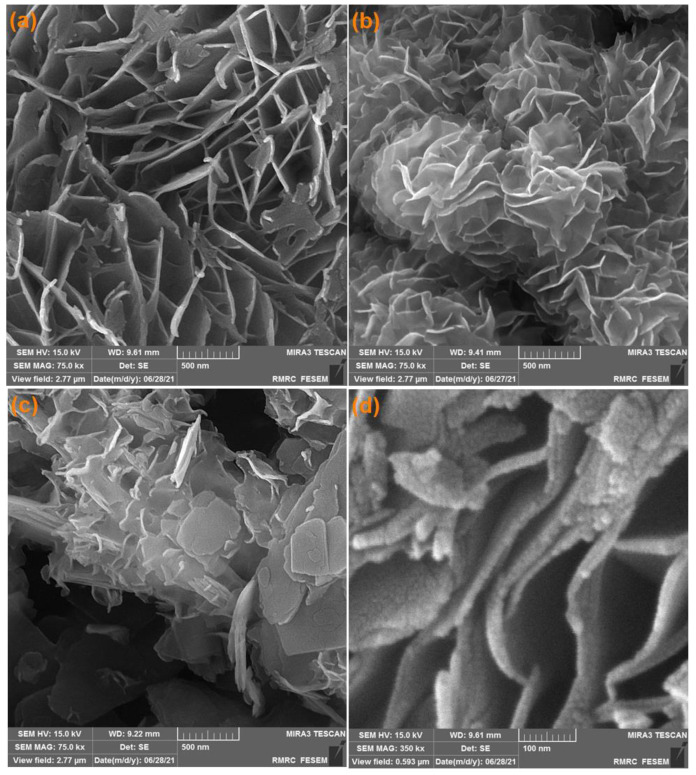
FE-SEM images of Ni-MOF nanosheets (**a**), MoS_2_ flower-like nanosheets (**b**), MoS_2_/Ni-MOF hybrid NSs (**c**,**d**).

The FE-SEM images captured from Ni-MOF (Figure 1a) shows approximately transparent 20-nanometer NSs and also well-defined two-dimensional layer of fabricated Ni-MOF. A flower-like MoS_2_ nanostructure constructed by the assembly of 2D NSs is shown in Figure 1b. The NSs cut with a smooth surface show an identical thickness and a distinct gap of the layers. Figure 1b illustrates the FE-SEM image captured from MoS_2_, highlighting a 13-nanometer nanosheet morphology. Moreover, the obtained MoS_2_/Ni-MOF hybrid NSs exhibit a hierarchical structure while retaining the 2D NS property (Figure 1c,d), so that the MoS_2_ NS surface is covered densely by Ni-MOF, leading to a relatively uneven surface.

**Figure 2:**
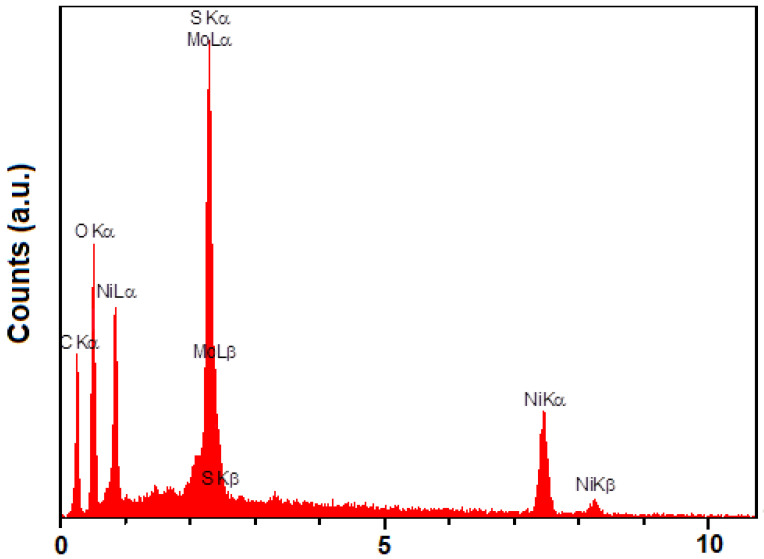
EDX spectrum of MoS2/Ni-MOF hybrid NSs.

Energy dispersive X-ray findings (Figure 2) illustrate only the presence of Mo, S, C, O, and Ni in the structure of MoS_2_/NiMOF hybrid NSs.

**Figure 3:**
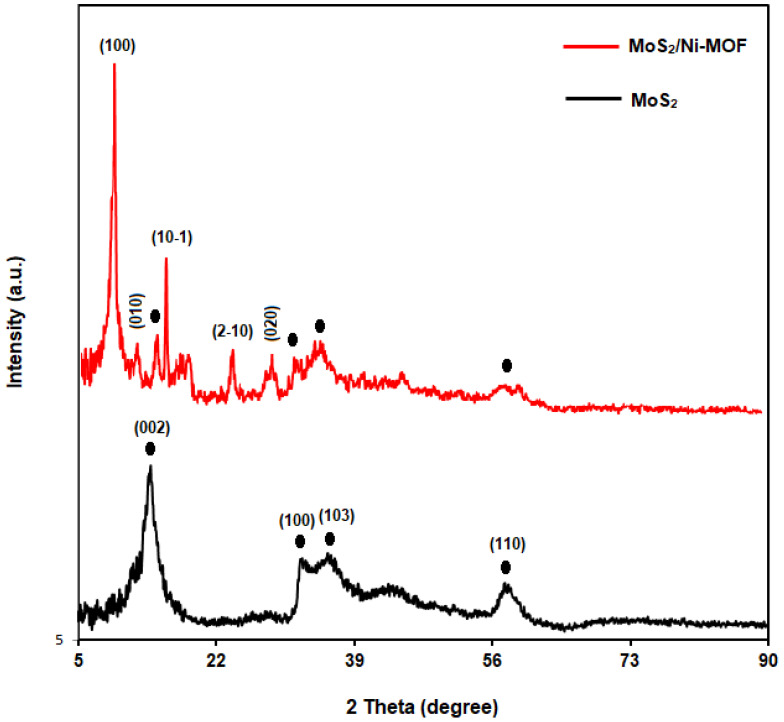
XRD patterns of MoS_2_ and MoS_2_/Ni-MOF hybrid NSs.

Figure 3 shows the XRD patterns captured for the pure MoS_2_ and MoS_2_/Ni-MOF hybrid NSs. Pure MoS_2_ curves exhibited high purity for the samples produced. The peaks appearing at 13.66°, 32.22°, 35.3° and 57.8° were indexed to planes of (002), (100), (103) and (110), sequentially [60]. Overall, the sharp diffraction peaks of MoS_2_ verifies a great crystallinity. MoS_2_/Ni-MOF hybrid NSs not only had the characteristic peaks related to MoS_2_, but also displayed clear related to Ni-MOF, corresponding to the planes of (100), (010), (10-1), (2-10), and (020) [61,62].

**Figure 4:**
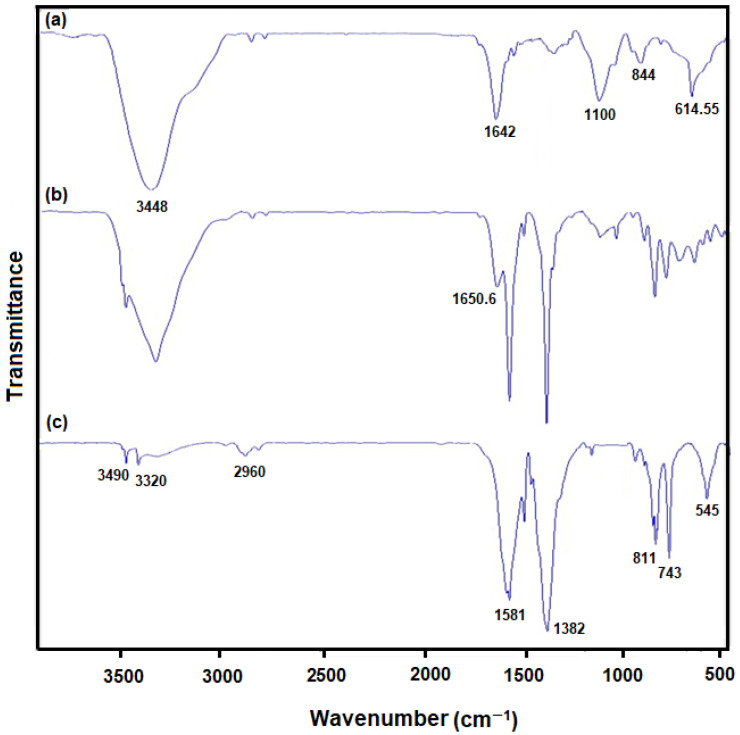
FT-IR patterns of (**a**) MoS_2_ flower-like nanosheets, (**b**) MoS_2_/Ni-MOF hybrid NSs, and (**c**) Ni-MOF nanosheets.

The composition of as-produced hybrid NSs was explored using the Fourier transform infrared spectroscopy (Figure 4). The FT-IR spectrum revealed the peaks at 614.55 and 884 cm^−1^ for MoS_2_ (Figure 4; curve a) respectively corresponding to Mo-S and S-S vibrations. The peaks formed at 1100, 1642 and 3448 cm^−1^ corresponded to hydroxyl stretching vibration resulting from water molecules absorbed [63,64]. The FT-IR spectrum obtained for Ni-MOF (Figure 4; curve c) showed strong peaks at 3490 and 3320 cm^−1^ corresponding to hydroxyl (−OH) stretching vibration, and at 2960, 811 and 743 cm^−1^ corresponding to aromatic units’ C–H bonds, as well as at 1382 and 1581 cm^−1^ respectively corresponding to terephthalic anions’ Vs(COO) and Vas(COO). The peak at 545 cm^−1^ corresponding to ν(Ni–O) verifies a metal–oxo bond between the terephthalic acid’s carboxylic group and Ni atoms [62]. When comparing to Ni-MOF and MoS_2_, a new peak appeared strongly at 1650.6 cm^−1^ related to MoS_2_/Ni-MOF hybrid NSs (Figure 4; curve b) because of an interplay of MoS_2_ with Ni-MOF, boosting C=C stretching vibration. Consequently, the MoS_2_/Ni-MOF hybrid NSs possess all absorption bands of MoS_2_ and Ni-MOF [62].

**Figure 5:**
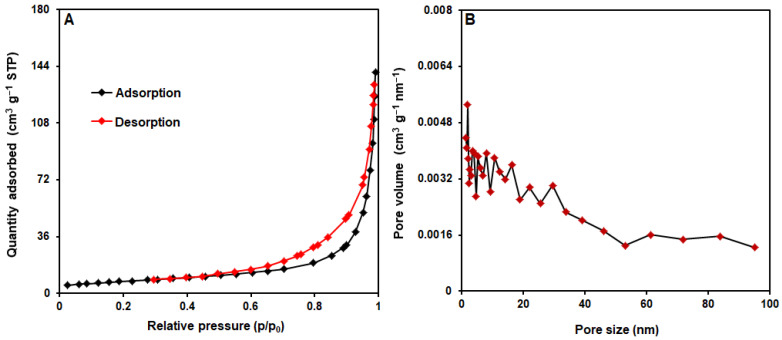
(**A**) N_2_ adsorption−desorption isotherms and (**B**) pore size distribution of MoS_2_/Ni-MOF hybrid NSs.

Figure 5 shows the calculation of MoS_2_@Ni-MOF pore size and surface areas based on nitrogen adsorption−desorption isotherms adopted from Barrett-Joyner-Halenda and Brunauer-Emmett-Teller methods [62,65]. In accordance with the graphical isotherm of typical H3 hysteresis loop in Figure 5A, MoS_2_/Ni-MOF hybrid NSs follows the type-IV isotherms. The accumulation of pores is evident based on the hysteresis loop of MoS_2_/Ni-MOF at relative high pressure (p/p_0_). The surface area of 27.19 m^2^/g was computed for the MoS_2_/Ni-MOF. According to the distribution of pore size in Figure 5B, the MoS_2_/Ni-MOF had the pore diameter of 1.85 nm.

### 3.2. Electrochemical Performance of 4-AP on the Surface of MoS_2_/Ni-MOF/SPGE

Research indicates that the pH of the electrolytes is one of the fundamental factors influencing the 4-AP response on the MoS_2_/Ni-MOF/SPGE. Hence, researchers cautiously used DPV in the pH ranges between 2.0 and 9.0 through 0.1 M PBS to determine the impact of the pH value on the electro-chemical behaviors of 4-AP. Analyses revealed that the peak current 4-AP of oxidation enhanced when pH value elevated to 7.0 but it was reduced with an increase in pH. For achieving higher sensitivity, we chose pH 7.0 as an optimal pH to electrochemically detect the 4-AP on the MoS_2_/Ni-MOF/SPGE.

**Figure 6:**
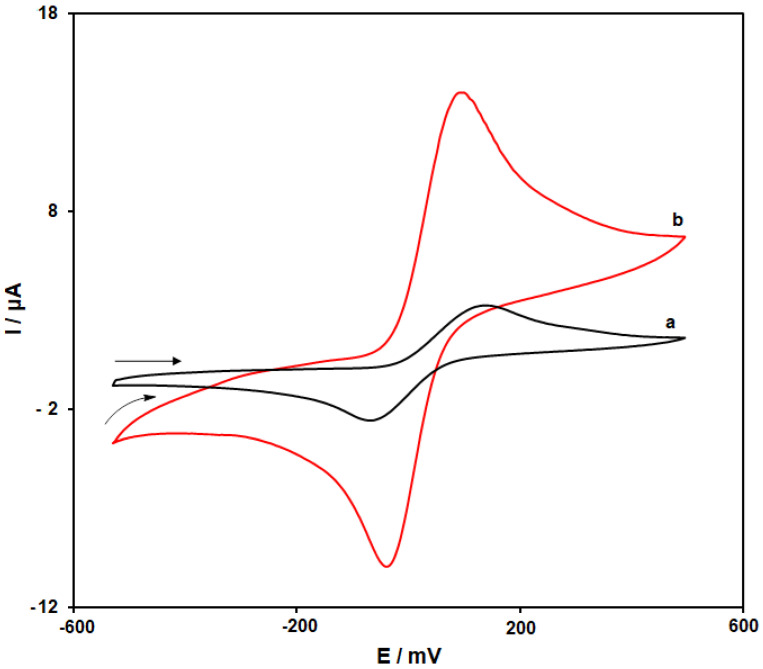
CVs of the bare SPGE (**a**) and MoS_2_/Ni-MOF/SPGE (**b**) in the 0.1 M PBS (pH = 7.0) with 200.0 µM 4-AP at the scanning rate of 50 mVs^−1^.

The CVs of 4-AP at the scanning rate of 50 mV s^−1^ in the 0.1 M PBS (pH = 7.0) on the bare SPGE (curve a) as well as the MoS_2_/Ni-MOF/SPGE (curve b) are depicted in Figure 6. As seen in Figure 6 (curve a), the cathodic and anodic peaks of 4-AP on the bare SPGE were observed at −70 and 145 mV, respectively, while the separation between peak potentials (ΔEp) was observed at 213 mV. Figure 6 (curve b) demonstrates the further enhancement of the 4-AP oxidation peak current on the MoS_2_/Ni-MOF/SPGE compared to that of the bare SPGE, negative shift of the anodic peak potential to 105 mV as well as positive shift of the cathodic peak potential to −35 mV. Furthermore, ΔEp of 4-AP on the MoS_2_/Ni-MOF/SPGE equaled 140 mV. Consequently, we found a considerable increase of the 4-AP redox peak currents on the MoS_2_/Ni-MOF/SPGE caused by acceptable conductivity and electrocatalytic feature of MoS_2_/Ni-MOF.

### 3.3. The Effect of the Scanning Rate

**Figure 7:**
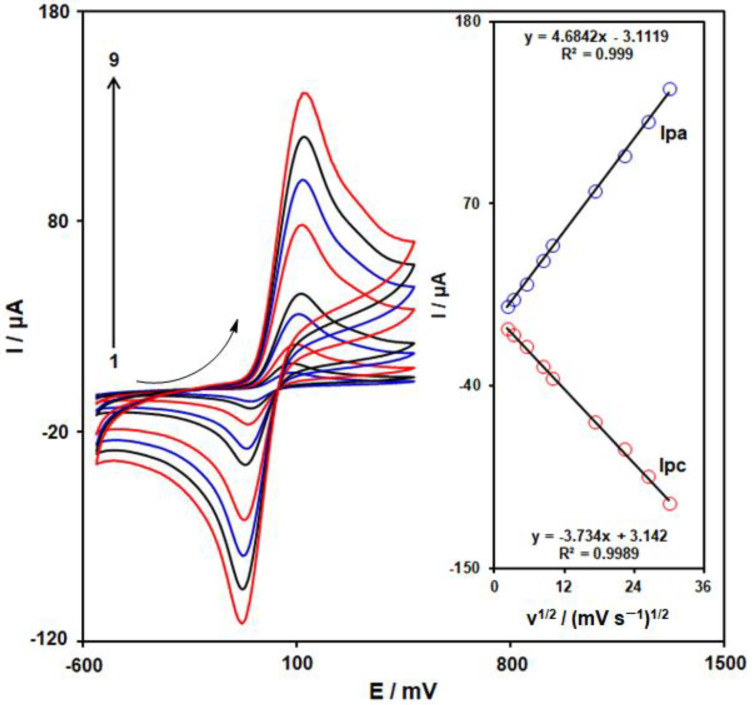
CVs observed on the MoS_2_/Ni-MOF/SPGE with 400 µM 4-AP in the 0.1 M PBS (pH = 7.0) at different scanning rates of 1–9: 5, 10, 30, 70, 100, 300, 500, 700, and 900 mV/s. Inset: Plot of the variation of I_pc_ and I_pa_ vs. υ^1/2^ for 4-AP electrooxidation.

We used CV in this step to determine the impact of the scanning rate on the redox reaction of 4-AP on the surface of MoS_2_/Ni-MOF/SPGE. Figure 7 represents the CV curves of 400.0 µM 4-AP on the MoS_2_/Ni-MOF/SPGE with diverse scanning rates from 5–900 mVs^−1^. Considering the figure, the cathodic and anodic peak currents (I_pc_, I_pa_) of 4-AP were elevated by enhancing the scanning rates. Therefore, values of I_pc_ and I_pa_ exhibit an acceptable linear correlation to the square root of the scanning rate (υ^1/2^) (see Inset in Figure 7). Thus, it could be concluded that the electrode reaction of 4-AP on the MoS_2_/Ni-MOF/SPGE would be a process controlled by diffusion.

### 3.4. Chronoamperometric Measurements

**Figure 8:**
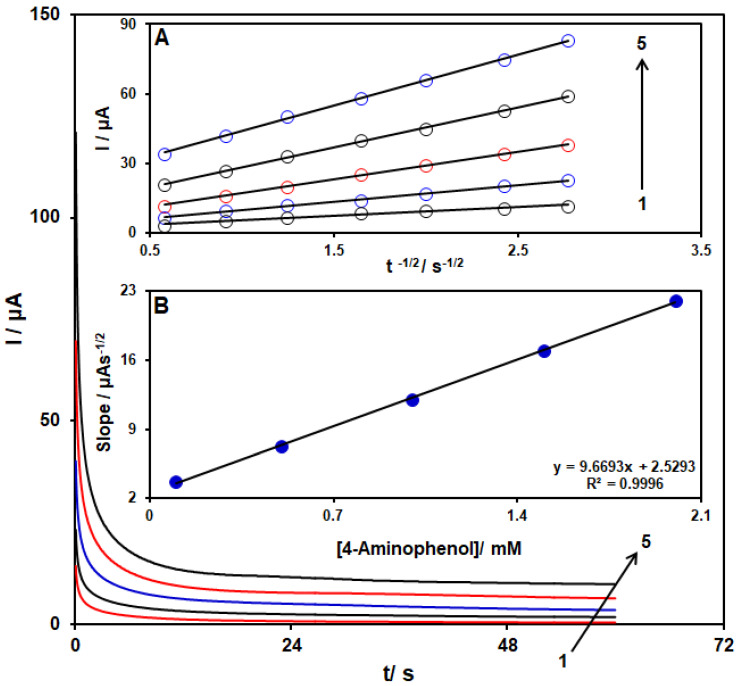
4-AP chronoamperograms with several concentrations (from 1 to 5: 0.1, 0.5, 1.0, 1.5, and 2.0 mM) on the MoS_2_/Ni-MOF/SPGE in the 0.1 M PBS (pH = 7). Inset (**A**). *I* vs. *t*^−1/2^ plot for 4-AP electro-oxidation achieved by chronoamperoms 1–5. Inset (**B**). The slope plot from the straight lines vs. level of 4-AP.

Chronoamperometry was employed to study the 4-AP electro-oxidation using a MoS_2_/Ni-MOF/SPGE (see Figure 8). Therefore, the potential of the working electrode was set at 0.14 V as the first-step potential to measure the chronoamperometry of several concentrations of 4-AP on the MoS_2_/Ni-MOF/SPGE sensor. In this way, the diffusion coefficient, *D*, of 4-AP in 0.1 M PBS was identified by chronoamperometric tests. Then, we applied the experimental plots of *I*p vs. *t*^−1/2^ with the best fits for several concentrations of 4-AP (see Figure 8A). Moreover, the slopes shown for the final straight lines were drawn vs. 4-AP concentrations (refer to Figure 8B) and thus *D* = 3.2 × 10^−5^ cm^2^/s by the Cottrell equation and the obtained slopes.

### 3.5. Calibration Plot and Detection Limit

**Figure 9:**
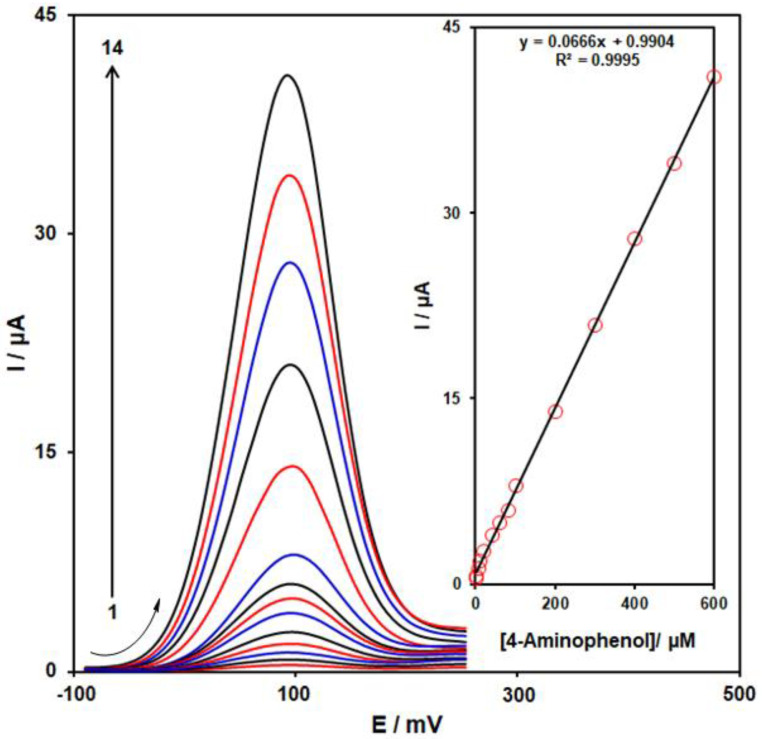
DPVs of 4-AP with diverse concentrations (from 1 to 14: 0.1, 1.0, 5.0, 10.0, 20.0, 40.0, 60.0, 80.0, 100.0, 200.0, 300.0, 400.0, 500.0, & 600.0 µM) on the MoS_2_/Ni-MOF/SPGE in 0.1 M PBS (pH = 7). Inset: linear relationship of the peak current with 4-AP concentration (from 0.1 μM to 600.0 μM).

According to the research design, we employed DPV at optimum conditions to measure several concentrations of 4-AP in the 0.1 M PBS (pH = 7.0) for evaluating the analytical functions of the MoS_2_/Ni-MOF/SPGE sensor. With regard to Figure 9 the increased peak current was observed as the concentration of 4-AP elevated from curve 1 to 14, reflecting a satisfactory linear correlation to the 4-AP concentration (as depicted in the inset of Figure 9) in ranges 0.1–600.0 μM, which followed the correlation equation of Ipa = 0.0666 C_4-AP_ + 0.9904 (R^2^ = 0.9995). It should be noted that LOD equaled 0.04 µM. Two-dimentional Ni-MOFs have shown special attributes such as high porosity and nano-sized thickness that predispose excellent electron transfer and rapid mass transport. Also, the synergistic effect between Ni-MOF and Mos_2_ increases the conductivity and electro-catalytic activity of the proposed MoS_2_/Ni-MOF/SPG sensor. The electro-analytical performance for 4-AP at MoS_2_/Ni-MOF/SPGE was compared with the other chemically modified electrodes (Table 1). These findings revealed the acceptable analytical function of MoS_2_/Ni-MOF/SPGE sensor for 4-AP detection.

**Table 1:** Comparison of various modified electrodes for the detection of 4- AP with MoS_2_/Ni-MOF/SPGE.

Electrochemical Sensor	Method	Linear Range	LOD	Real Samples	Ref.
Chitosan-Au nanoparticles-Pd-reduced graphene oxide nanohybrid/glassy carbon electrode	DPV	1.0–300.0 μM	0.12 μM	Water	[66]
Hemin-molecularly imprinted polymer/glassy carbon electrode	Amperometric	10.0–90.0 μM	3.0 μM	Tap and river water	[67]
Graphene/hydroxyapatite nanocomposite/glassy carbon electrode	Square wave voltammetry	0.1–425.0 μM	0.29 μM	Tap water	[68]
Graphene–chitosan composite/glassy carbon electrode	DPV	0.2–550.0 μM	0.057 μM	River water, Lake water, Waste water, and Tap water	[69]
Graphene–polyaniline nanocomposite/glassy carbon electrode	DPV	0.2–100.0 μM	0.065 μM	-	[70]
MoS_2_/Ni-MOF/SPGE	DPV	0.1–600.0 μM	0.04 μM	Acetaminophen tablet and tap water	This Work

### 3.6. Determination of 4-AP in Presence ACAP

**Figure 10:**
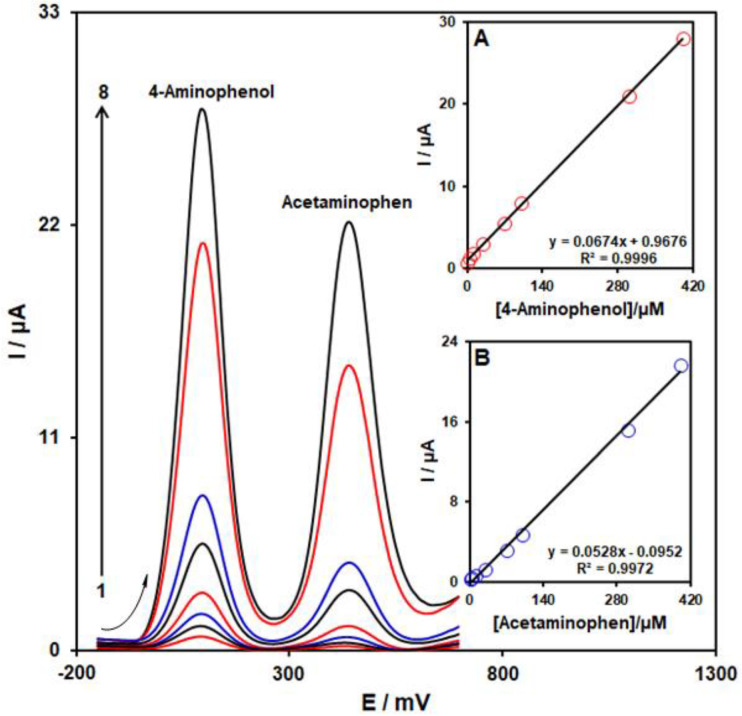
DPVs of 4-AP and ACAP with several concentrations (from 1 to 8: 1.0 + 1.0, 5.0 + 5.0, 10.0 + 10.0, 30.0 + 30.0, 70.0 + 70.0, 100.0 + 100.0, 300.0 + 300.0, and 400.0 + 400.0 µM) on the MoS_2_/Ni-MOF/SPGE in 0.1 M PBS (pH = 7). Inset: (**A**) the peak current plot as the function of concentration of 4-AP and (**B**) the peak current plot as the function of ACAP concentration.

Upon the creation of the optimized conditions, as a result of its more acceptable resolution and greater current sensitivity, DPV was employed for the quantitative simultaneous detection of 4-AP and ACAP. Figure 10 presents DPVs of various concentrations of 4-AP and ACAP on the MoS_2_/Ni-MOF/SPGE surface. Considering Figure 10, we observe 2 complete oxidation peaks at 100 mV and 440 mV for 4-AP and ACAP. Moreover, 4-AP and ACAP concentrations exhibit an acceptable linear relationship to the respective oxidation peak currents in ranges between 1.0 µM and 400.0 µM (see Insets A and B). In addition, sensitivity to 4-AP in the case of the presence and absence of ACAP equaled 0.0674 µA/µM (see Figure 10 Inset A) and 0.0666 µA/µM (see inset of Figure 9), respectively. Hence, we inferred that it is possible to use MoS_2_/Ni-MOF/SPGE successfully for the simultaneous detection of 4-AP and ACAP with higher sensitivity and better selectivity.

### 3.7. ACAP and 4-AP Detection in the Real Samples

Since our research aimed for assessing the new approach practically, we employed MoS_2_/Ni-MOF/SPGE for ACAP and 4-AP detection in the acetaminophen tablet and tap water samples with the standard addition method. Hence, each measurement was iterated give times in similar conditions. See Table 2 for more results. As seen in the table, recovery of ACAP and 4-AP equaled 97.0–103.3% and 96.2–104.3%, indicating the possible use of our electrochemical sensor to detect ACAP and 4-AP in the real samples.

## 4. Conclusions

We produced new MoS_2_/Ni-MOF hybrid nanosheets as the modifier for screen-printed graphite electrode surface modification to fabricate an ultra-sensitive sensor for the electrochemical detection of 4-AP. Findings presented an admirable electro-catalytic behavior for as-developed hybrid nanosheets towards the oxidation of 4-AP owing to commendable mass transfer, abundant active sites, impressive conductivity and huge surface area. As-developed MoS_2_/Ni-MOF/SPGE had a broad LDR (0.1–600.0 μM) and a narrow LOD towards the oxidation of 4-AP. Moreover, 4-AP and ACAP were electrochemically detected concurrently on the modified electrode surface, with a peak potential separation of 340 mV. In addition, the analysis of real specimens such as tap water sample as well as a commercial sample (acetaminophen tablets) illuminated the successful applicability of the as-developed sensor in determining ACAP and 4-AP, with an impressive recovery rate.

## Figures and Tables

**Table 2:** Findings for 4-AP and ACAP detection in real samples using MoS_2_/Ni-MOF/SPGE. All concentrations are in µM (n = 5).

Sample	Spiked		Found		Recovery (%)		R.S.D. (%)	
	4-AP	ACAP	4-AP	ACAP	4-AP	ACAP	4-AP	ACAP
Acetaminophen tablets	0	0	-	4.0	-	-	-	3.5
	5.0	2.0	4.9	6.2	98.0	103.3	1.9	2.9
	6.0	3.0	6.1	6.9	101.7	98.6	2.7	2.1
	7.0	4.0	7.3	7.9	104.3	98.7	3.2	1.8
	8.0	5.0	7.7	9.2	96.2	102.2	2.8	2.2
Tap water	0	0	-	-	-	-	-	-
	5.5	6.0	5.6	5.9	101.8	98.3	2.1	3.6
	6.5	8.0	6.3	8.2	97.0	102.5	3.0	2.3
	7.5	10.0	7.6	9.7	101.3	97.0	1.9	2.8
	8.5	12.0	8.4	12.2	98.8	101.7	2.4	1.8

## Data Availability

The data presented in this study are available upon request from the corresponding authors.
